# Impacts of fixation processing workflows on the volatile and non-volatile metabolomic profiles of Gougunao green tea

**DOI:** 10.3389/fnut.2026.1886403

**Published:** 2026-07-24

**Authors:** Jie Zhou, Wentao Li, Jinyun Peng, Yifan Cai, Junjie Lu, Yongfa Guo, Shuaiwen Yin, Xiaojin Liang, Huimin Sun, Xinjun Liao

**Affiliations:** 1 School of Life Sciences, Jinggangshan University, Ji’an, China; 2 Key Laboratory of Jiangxi Province for Biological Invasion and Biosecurity, School of Life Sciences, Jinggangshan University, Ji’an, China; 3 Key Laboratory of Jiangxi Province for Functional Biology and Pollution Control in Red Soil Regions, School of Life Sciences, Jinggangshan University, Ji’an, China; 4 College of Agriculture and Biotechnology, Zhejiang University, Hangzhou, China; 5 Jiangxi Tongzhou Green Food Development Co., Ltd., Ji’an, China

**Keywords:** fixation processing workflow, Gougunao tea, HS-SPME-GC-MS, OPLS-DA, UHPLC-MS/MS, volatile organic compounds

## Abstract

Fixation is a central thermal step in green tea processing, but Gougunao tea uses a distinctive two-stage fixation and rolling workflow that has not been systematically evaluated at the metabolomic level. Here, four fixation processing workflows were compared: fully mechanical fixation (M1), mechanical–manual hybrid fixation (M2), manual–mechanical hybrid fixation (M3), and fully manual fixation (M4). An integrated UHPLC-MS/MS and HS-SPME-GC-MS strategy was used to profile non-volatile and volatile metabolites. In total, 2,105 non-volatile metabolic features and 1,066 volatile metabolites were putatively annotated. PCA showed clear workflow-associated separation in both LC-MS and GC-MS datasets, while OPLS-DA supported pairwise discrimination among most comparisons. Differential screening using FDR < 0.05 and |log₂FC| > 1 identified 16–183 differential non-volatile metabolites and 0–26 differential volatile metabolites across the six pairwise comparisons. Non-volatile differences were mainly distributed among lipids and lipid-like molecules, organoheterocyclic compounds, organic acids and derivatives, benzenoids, and phenylpropanoids and polyketides, indicating broad changes in metabolite pools related to tea taste formation, phenolic transformation, lipid-derived reactions, and secondary metabolism. Representative taste-associated compounds, including amino acids, catechins, methylxanthines, phenolic acids, and theaflavin-related metabolites, showed workflow-dependent abundance patterns. For volatile compounds, FDR-significant markers and relative odor activity value (rOAV) ranking highlighted aldehydes, esters, alcohols, ketones, and sulfur-containing compounds as candidate aroma-related volatiles. These results provide metabolomic evidence that different fixation processing workflows are associated with distinct chemical profiles in Gougunao green tea, while sensory validation remains necessary to confirm their direct quality implications.

## 1 Introduction

Tea (*Camellia sinensis*) is one of the most widely consumed non-alcoholic beverages worldwide, and green tea is valued for its fresh aroma, characteristic bitterness and astringency, umami-related taste, and health-associated phytochemicals (1). These sensory and chemical properties are strongly shaped by processing. Among the processing steps of green tea, fixation is a decisive thermal treatment because it rapidly inactivates oxidative enzymes, limits enzymatic browning, and initiates heat-driven chemical transformations that affect both non-volatile taste-related compounds and volatile aroma-related compounds (2, 3). Recent metabolomic studies have shown that tea processing can reshape amino acids, catechins, flavonoids, phenolic acids, lipids, glycosides, and aroma precursors, providing a chemical basis for understanding quality formation during tea manufacture (4–6).

Increasing evidence indicates that the metabolic effects of tea processing depend not only on a single step, but also on the way multiple operations are combined. In green tea, fixation conditions and fixation methods have been linked to changes in both non-volatile and volatile metabolites related to flavor development (2, 3, 7). Other processing steps, including spreading, rolling, and drying, can also influence the accumulation or transformation of quality-related metabolites (8–10). These studies suggest that green tea quality is shaped by integrated processing workflows rather than by isolated technological parameters alone.

Metabolomics has become an important approach for characterizing tea-processing-induced chemical variation. Reviews and large-scale studies have highlighted its value for linking processing methods, geographical origins, harvest seasons, and chemical composition in tea (4, 6). More recent studies have further connected metabolomic changes with measurable taste-related outcomes. For instance, metabolomics combined with electronic tongue analysis has been used to track taste-metabolite evolution during industrial black tea processing (11). In Duyun Maojian green tea, widely targeted metabolomics, sensory evaluation, and electronic tongue analysis showed that fixation and drying were critical stages affecting non-volatile metabolites and taste profiles (12). These studies provide useful references for interpreting taste-associated metabolites, but comparable evidence remains limited for regional green teas with distinctive multi-step fixation and rolling workflows.

Gougunao tea is a geographical indication green tea from Jiangxi Province, China, and is characterized by a twisted shape and a traditional manufacturing process involving two rounds of fixation and rolling. A recent metabolomic study distinguished Gougunao tea from different geographical origins using metabolomics combined with sensory evaluation, but it did not examine how different fixation processing workflows affect the volatile and non-volatile metabolome of Gougunao tea (13). In local production, mechanical fixation, manual fixation, and mechanical–manual hybrid workflows coexist. These workflows differ not only in the order of mechanical and manual fixation, but also in fixation temperature, fixation duration, and matched rolling time. Therefore, they should be treated as integrated fixation–rolling workflows rather than as fixation sequence alone. Although machine and manual fixation have been compared in Xihu Longjing tea, that work focused on another green tea type and did not jointly profile volatile and non-volatile metabolites in Gougunao tea (14).

To address this gap, the present study compared four Gougunao tea fixation processing workflows: fully mechanical fixation, mechanical–manual hybrid fixation, manual–mechanical hybrid fixation, and fully manual fixation. UHPLC-MS/MS was used to profile non-volatile metabolic features, and HS-SPME-GC-MS was used to characterize volatile metabolites. The objectives were to compare the global non-volatile and volatile metabolomic profiles of Gougunao tea produced by these workflows, identify FDR-significant differential metabolites, summarize their chemical-class distributions, and characterize taste-associated non-volatile metabolites and candidate aroma-related volatile markers. Together, this integrated metabolomic strategy was designed to clarify how different thermal-mechanical fixation histories reshape quality-related chemical profiles in Gougunao green tea.

## 2 Materials and methods

### 2.1 Experimental materials

The fresh leaves of Gougunao tea (*Camellia sinensis* cv. **“**Gougunao No. 2**”**) were collected from the Gougunao Tea Garden in Suichuan County, Jiangxi Province, China (26°15′N, 114°10′E). The leaves were harvested in mid-April according to a one-bud-two-leaf plucking standard. HPLC-grade methanol and acetonitrile were obtained from Fisher Scientific (USA), and formic acid was obtained from TCI (Japan). L-2-chlorophenylalanine was purchased from Aladdin (China). For GC**-**MS analysis, analytical-grade sodium chloride (NaCl) was obtained from China National Pharmaceutical Group. All four processing workflows were applied to a single homogeneous batch of fresh leaves to minimize raw-material variation among treatments.

### 2.2 Processing methods for Gougunao tea

Four processing workflows involving different combinations of mechanical and manual fixation were compared to evaluate workflow-associated changes in the metabolomic profile of Gougunao tea. Mechanical fixation followed a high-temperature short-time condition (220 ± 2 °C, 3 min), whereas manual fixation followed a lower-temperature longer-duration condition (180 ± 2 °C, 15–20 min). Rolling duration was matched to the fixation mode: 5 ± 0.5 min after mechanical fixation and 10 ± 0.5 min after manual fixation. These processing parameters were adopted from the routine production protocol of the collaborating Gougunao tea producer and were confirmed in pilot runs before formal sampling to ensure stable operation. Therefore, M1–M4 should be interpreted as integrated processing workflows that differed in fixation mode, temperature, duration, and matched rolling time, while spreading, drying, equipment, and operator conditions were kept consistent.

#### 2.2.1 Method 1: full mechanical fixation (M1)

Fresh tea leaves → spreading (leaf layer thickness: 2 cm, 4–8 h at 20–25 °C, RH 84.71 ± 0.5%) → primary mechanical fixation (220 ± 2 °C, 3 min; Model 6CST-50 drum fixation machine, Zhejiang Shangyang Machinery Co., Ltd., China) → first rolling (5 ± 0.5 min) → secondary mechanical fixation (220 ± 2 °C, 3 min) → secondary rolling (5 ± 0.5 min) → drying (90 ± 1 °C, 60 min) → dried tea.

#### 2.2.2 Method 2: mechanical–manual hybrid fixation (M2)

Fresh tea leaves → spreading (leaf layer thickness: 2 cm, 4–8 h at 20–25 °C, RH 84.71 ± 0.5%) → primary mechanical fixation (220 ± 2 °C, 3 min; Model 6CST-50 drum fixation machine, Zhejiang Shangyang Machinery Co., Ltd., China) → first rolling (5 ± 0.5 min) → secondary manual fixation (180 ± 2 °C, 15 min) → secondary rolling (10 ± 0.5 min) → drying (90 ± 1 °C, 60 min) → dried tea.

#### 2.2.3 Method 3: manual–mechanical hybrid fixation (M3)

Fresh tea leaves → spreading (leaf layer thickness: 2 cm, 4–8 h at 20–25 °C, RH 84.71 ± 0.5%) → primary manual fixation (180 ± 2 °C, 20 min) → first rolling (10 ± 0.5 min) → secondary mechanical fixation (220 ± 2 °C, 3 min; Model 6CST-50 drum fixation machine, Zhejiang Shangyang Machinery Co., Ltd., China) → secondary rolling (5 ± 0.5 min) → drying (90 ± 1 °C, 60 min) → dried tea.

#### 2.2.4 Method 4: full manual fixation (M4)

Fresh tea leaves → spreading (leaf layer thickness: 2 cm, 4–8 h at 20–25 °C, RH 84.71 ± 0.5%) → primary manual fixation (180 ± 2 °C, 20 min) → first rolling (10 ± 0.5 min) → secondary manual fixation (180 ± 2 °C, 15 min) → secondary rolling (10 ± 0.5 min) → drying (90 ± 1 °C, 60 min) → dried tea.

Each workflow was prepared in three independent processing replicates from the same homogeneous fresh-leaf batch.

### 2.3 Metabolite extraction

For LC-MS analysis, tea infusion samples stored at −80 °C were thawed on ice and vortexed for 30 s. A 2 mL aliquot of each sample was transferred into a 15 mL centrifuge tube, rapidly frozen in liquid nitrogen, and lyophilized. The dried residue was reconstituted in 1 mL of ice-cold methanol:acetonitrile (1:1, v/v), vortexed for 30 s, and incubated at −20 °C for 30 min. After centrifugation at 12,000 rpm and 4 °C for 10 min, 850 μL of the supernatant was collected and vacuum-dried. The residue was then reconstituted in 150 μL of 50% (v/v) aqueous methanol containing 5 mg/L L-2-chlorophenylalanine as an internal standard to monitor sample preparation consistency and instrumental response stability. The mixture was vortexed for 30 s and centrifuged again at 12,000 rpm and 4 °C for 10 min. The supernatant was filtered through a 0.22 μm hydrophilic membrane and transferred into autosampler vials. Quality control (QC) samples were prepared by pooling 10–20 μL of filtrate from each individual sample. The pooled QC sample was vortexed and centrifuged under the same conditions and was used to monitor instrument stability and data reproducibility throughout the analytical sequence.

For GC-MS volatile analysis, tea infusion samples stored at −80 °C were thawed on ice and vortexed. A 1 mL aliquot of each sample was transferred into a headspace vial, followed by the addition of 1 mL of saturated aqueous NaCl and 20 μL of internal standard solution (10 mg/L 3-hexanone-2,2,4,4-d4). Automated headspace solid-phase microextraction (HS-SPME) was then performed before GC-MS injection as described below.

### 2.4 Chromatography and mass spectrometry methods

#### 2.4.1 Ultra-high-performance liquid chromatography–tandem mass spectrometry

LC-MS/MS analysis was conducted on a Thermo Vanquish Flex UHPLC system (Thermo Fisher Scientific, Waltham, MA, United States) coupled to an Orbitrap Exploris 120 high-resolution mass spectrometer. Chromatographic separation was performed on an ACQUITY UPLC HSS T3 column (100 Å, 1.8 μm, 2.1 mm × 100 mm) maintained at 40 °C, with a flow rate of 0.4 mL/min. Mobile phase A was 0.1% (v/v) formic acid in water, and mobile phase B was 0.1% (v/v) formic acid in acetonitrile. The gradient program was as follows: 0–1.0 min, 5% B; 1.0–4.7 min, linear increase to 95% B; 4.7–6.0 min, 95% B; 6.0–6.1 min, decrease to 5% B; and 6.1–8.5 min, 5% B. The autosampler temperature was maintained at 8 °C, and the injection volume was 2 μL.

Mass spectrometric detection was performed using a heated electrospray ionization (HESI) source in both positive and negative ion modes, with spray voltages of +3.5 kV and −3.0 kV, respectively. Sheath gas and auxiliary gas flows were set to 40 and 10 arbitrary units, respectively. The capillary temperature was 320 °C, and the auxiliary gas heater temperature was 300 °C. Full-scan MS1 spectra were acquired over an m/z range of 70–1,000 at a resolution of 60,000, with an AGC target set to Standard and a maximum injection time of 100 ms. Data-dependent MS/MS acquisition was performed for the top four precursor ions using higher-energy collisional dissociation (HCD) at a normalized collision energy (NCE) of 30%, with an MS/MS resolution of 15,000 and a dynamic exclusion window of 4 s. Before formal sample analysis, 2–4 QC injections were performed to condition the instrument. During the analytical sequence, one QC sample was injected every 5–10 experimental samples to monitor instrument stability and analytical repeatability.

Non-volatile metabolite annotation was performed using MS-DIAL (version 4.9.221218) against the PSNGM database. This database integrates an in-house spectral library and public resources, including mzCloud, LIPID MAPS, HMDB, MoNA, NIST_2020_MSMS, and AI-predicted MS/MS spectral libraries. Candidate annotations were assigned based on MS1 accurate mass and MS/MS spectral matching, with MS1 and MS2 tolerances set to 0.01 Da and 0.05 Da, respectively. The MS-DIAL total score was used as the integrated identification score, and only features with a total score ≥70 were retained as annotated features. Candidate annotations were further checked using adduct information, isotope patterns, and fragment plausibility to reduce false-positive assignments. Because authentic standards were not available for most compounds, all non-volatile annotations listed in Supplementary Table S1 were reported as MSI Level 2 putatively annotated metabolic features. No MSI Level 1 confirmation was claimed.

#### 2.4.2 Headspace solid-phase microextraction gas chromatography–mass spectrometry

Volatile metabolites were extracted using a CTC Analytics AG SPME Arrow system equipped with an Agilent SPME Arrow fiber (120 μm DVB/CWR/PDMS). Before analysis, the fiber was pre-conditioned at 250 °C for 5 min in a CTC Analytics AG Fiber Conditioning Station. Samples were incubated at 60 °C with agitation for 5 min, followed by static headspace extraction for 15 min. The extracted analytes were thermally desorbed in the GC inlet at 250 °C for 5 min.

GC-MS analysis was performed on an Agilent 8,890 gas chromatograph coupled to an Agilent 7000D triple quadrupole mass spectrometer (Agilent Technologies, United States). Separation was achieved on a DB-5MS capillary column (30 m × 0.25 mm × 0.25 μm). Helium was used as the carrier gas at a constant flow rate of 1.2 mL/min. The injection port temperature was maintained at 250 °C, and the solvent delay was set to 3.5 min. The oven temperature program was as follows: 40 °C for 3.5 min; ramp to 100 °C at 10 °C/min; ramp to 180 °C at 7 °C/min; ramp to 280 °C at 25 °C/min; and hold at 280 °C for 5 min.

Mass spectrometric detection was performed in electron ionization mode at 70 eV. The ion source, quadrupole, and transfer line temperatures were set to 230 °C, 150 °C, and 280 °C, respectively. Data were acquired in time-scheduled selected ion monitoring (SIM) mode for library-based volatile profiling.

Volatile compound annotation was supported by an in-house database containing retention times, retention indices where available, standard compound spectra, literature information, and characteristic quantitative and qualitative ions. For each metabolite, one quantifier ion and two to three qualifier ions were monitored. A compound was annotated when its retention time and characteristic ion profile matched the database reference after background subtraction. Peak areas of quantifier ions were used for relative quantification, with the internal standard used to monitor analytical stability and signal variation.

### 2.5 Data preprocessing and statistical analysis

Raw LC-MS/MS data were imported into MS-DIAL (RIKEN, Japan; version 4.9.221218) for peak detection, alignment, filtering, gap filling, and metabolite annotation. Features not detected in more than 50% of QC samples were removed. Missing values were imputed using the MS-DIAL gap-filling algorithm, and normalized peak areas were exported for downstream analysis. For the revised annotation-based LC-MS/MS analyses and figure generation, annotated features with QC RSD > 30% were further excluded.

Raw GC-MS data acquired in SIM mode were processed using Agilent MassHunter software. Peak detection, integration, and relative quantification were performed based on predefined retention times, quantifier ions, and qualifier ions from the in-house volatile database. Non-detected peaks in the GC-MS peak-area matrix were replaced with one-fifth of the minimum positive value for the corresponding metabolite across all samples. Metabolites with QC CV ≥ 50% were removed, and the resulting analysis-ready GC-MS dataset was used for downstream analysis. Estimated relative odor activity values (rOAVs) were calculated as the ratio of relative volatile abundance to the corresponding odor threshold when threshold information was available.

The resulting LC-MS/MS and GC-MS peak-area matrices were further processed in R (version 4.1.2; R Foundation for Statistical Computing, Vienna, Austria). Prior to multivariate analysis, data were log₂-transformed and mean-centered. For OPLS-DA, LC-MS/MS data were Pareto-scaled to balance the contribution of high- and low-abundance features, whereas GC-MS data were analyzed after mean-centering alone to preserve the variance structure of the SIM-based relative intensity profiles. For PCA, both LC-MS/MS and GC-MS datasets were scaled to unit variance.

Principal component analysis (PCA) and orthogonal partial least squares discriminant analysis (OPLS-DA) were performed using the ropls package in R. OPLS-DA model performance was summarized using R^2^X, R^2^Y, and Q^2^ values. Potential overfitting was assessed using 200-iteration permutation tests by comparing the original Q^2^ values with the permuted Q^2^ distributions and regression intercepts. The OPLS-DA results were used to evaluate supervised group discrimination.

For pairwise differential analysis, fold change was calculated between groups, and statistical significance was evaluated using Student’s *t*-test. *p*-values were adjusted using the Benjamini-Hochberg procedure to control the false discovery rate (FDR). For the revised differential-metabolite analysis and main-figure generation, metabolites with |log₂FC| > 1 and FDR < 0.05 were considered significantly differential. VIP scores from OPLS-DA were used only as auxiliary multivariate information and were not used as mandatory screening criteria.

## 3 Results

### 3.1 Global metabolomic profiles differed among fixation processing workflows

**Figure 1:**
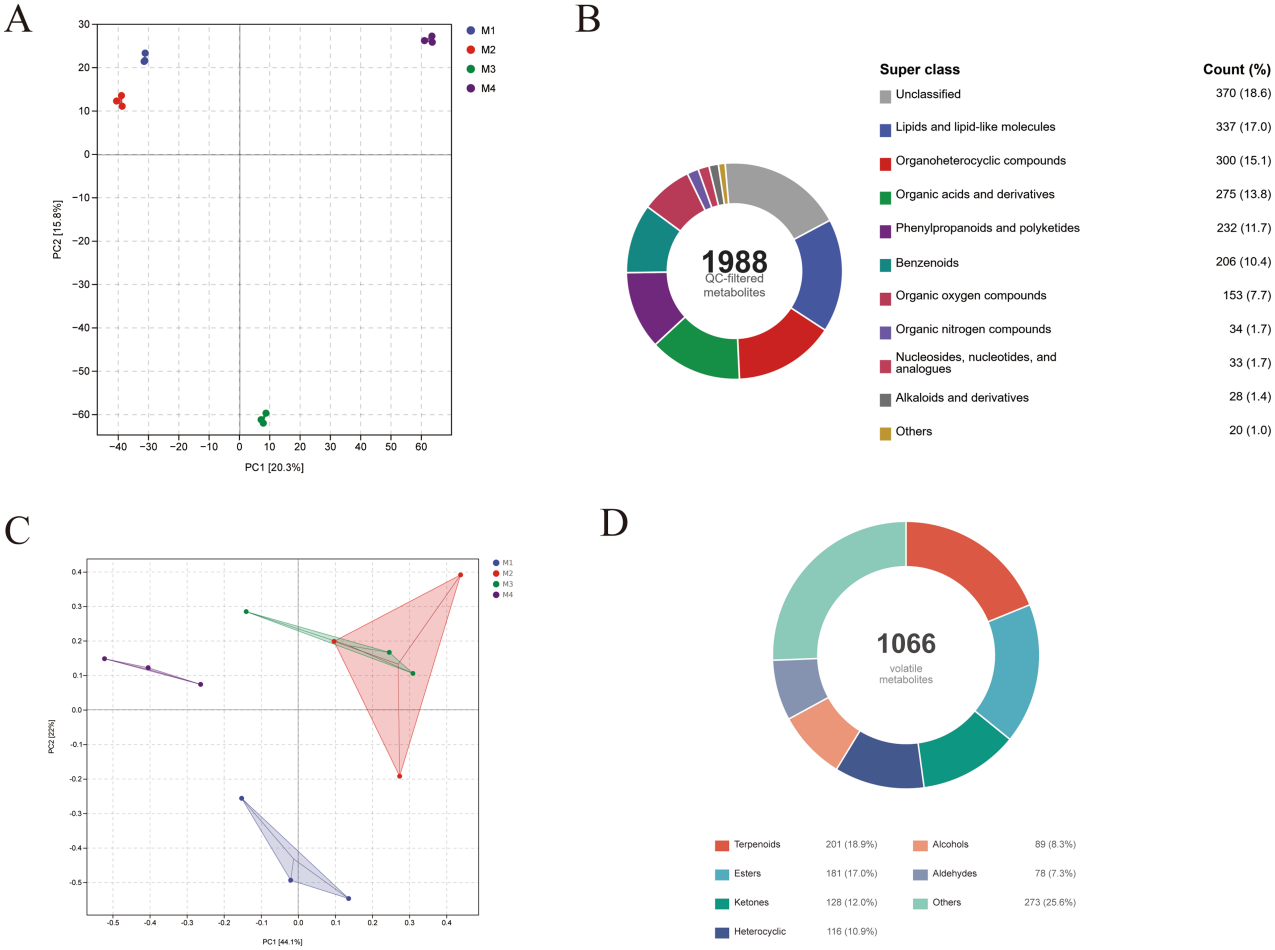
Global non-volatile and volatile metabolomic profiles of Gougunao green tea produced by four fixation processing workflows. **(A)** PCA score plot based on QC-filtered LC-MS features. **(B)** Super_Class distribution of QC-filtered non-volatile metabolites, including the unclassified fraction. **(C)** PCA score plot based on the GC-MS volatile dataset. **(D)** Chemical-class distribution of volatile metabolites after excluding the internal standard. M1, fully mechanical fixation; M2, mechanical–manual hybrid fixation; M3, manual–mechanical hybrid fixation; M4, fully manual fixation.

To evaluate the global chemical effects of different fixation processing workflows, non-volatile and volatile metabolomic profiles were examined using UHPLC-MS/MS and HS-SPME-GC-MS. In the LC-MS dataset, 2,105 non-volatile metabolic features were putatively annotated and reported in Supplementary Table S1. After quality-control filtering, 1,988 annotated features were retained for downstream analysis. These features included an unclassified fraction (370, 18.6%); among the Super_Class-annotated features, lipids and lipid-like molecules, organoheterocyclic compounds, organic acids and derivatives, phenylpropanoids and polyketides, benzenoids, and organic oxygen compounds represented the major groups (Figure 1B).

For the GC-MS dataset, Supplementary Table S2 contained 1,067 rows, including one internal standard. After excluding the internal standard, 1,066 volatile metabolites were used for subsequent analysis. These volatile compounds were classified into 15 chemical classes. Terpenoids, esters, ketones, heterocyclic compounds, alcohols, and aldehydes were the dominant categories, whereas the remaining minor classes were grouped as others for visualization (Figure 1D).

Unsupervised PCA based on 36,103 QC-filtered LC-MS features showed separation among the four processing workflows, with PC1 and PC2 explaining 20.3 and 15.8% of the total variance, respectively (Figure 1A). PCA of the GC-MS volatile dataset also separated the four groups, with PC1 and PC2 explaining 44.1 and 22.0% of the total variance, respectively (Figure 1C). These results indicate that the four fixation processing workflows produced distinguishable non-volatile and volatile chemical profiles.

**Figure 2:**
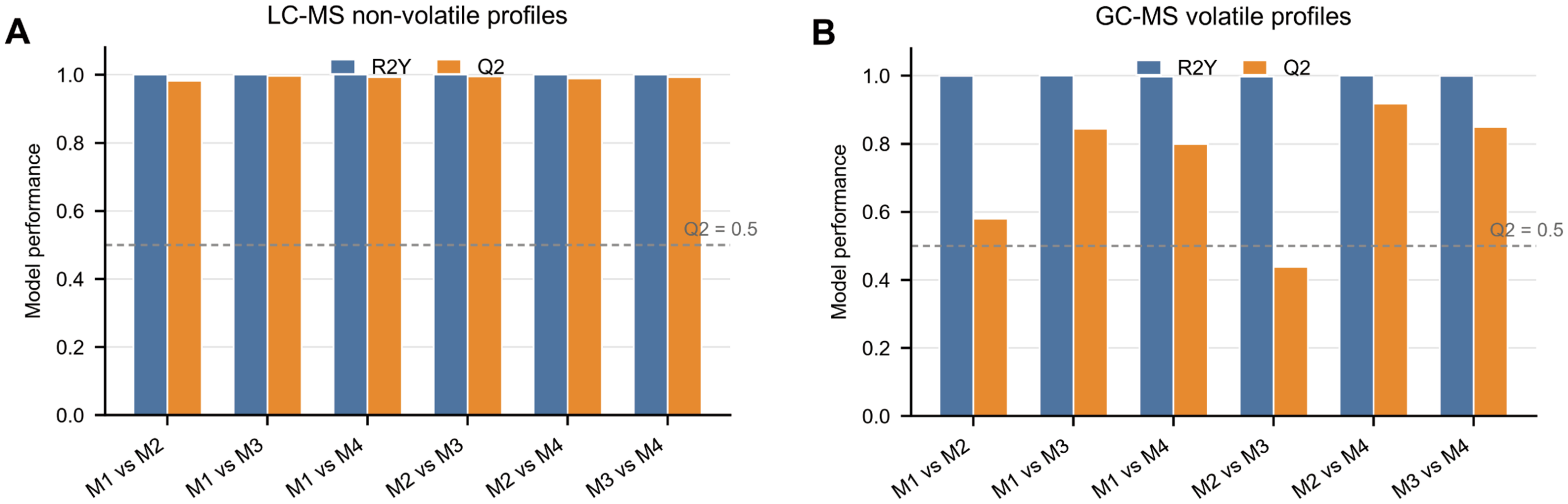
OPLS-DA model performance for pairwise comparisons among the four fixation processing workflows. **(A)** R^2^Y and Q^2^ summary of six pairwise OPLS-DA models based on the LC-MS dataset. **(B)** R^2^Y and Q^2^ summary of six pairwise OPLS-DA models based on the GC-MS dataset. Full OPLS-DA score plots and permutation tests are provided in Supplementary Figures S1–S4. The OPLS-DA models were used to evaluate supervised group discrimination.

OPLS-DA was then used to assess pairwise discrimination among the four groups. In the LC-MS dataset, the six pairwise models showed high R^2^Y values and Q^2^ values ranging from 0.983 to 0.997 (Figure 2A). In the GC-MS dataset, R^2^Y values ranged from 0.997 to 1.000, and Q^2^ values ranged from 0.438 to 0.918 (Figure 2B). The M2 vs. M3 volatile comparison showed the lowest Q^2^ value, consistent with the relatively small volatile difference between the two hybrid workflows. Full OPLS-DA score plots and permutation tests are provided in Supplementary Figures S1–S4. Considering the limited number of processing replicates, the OPLS-DA models were used as supervised discrimination evidence rather than as independent proof of biological causality.

### 3.2 Differential non-volatile metabolites were associated with taste- and secondary-metabolism-related classes

**Figure 3:**
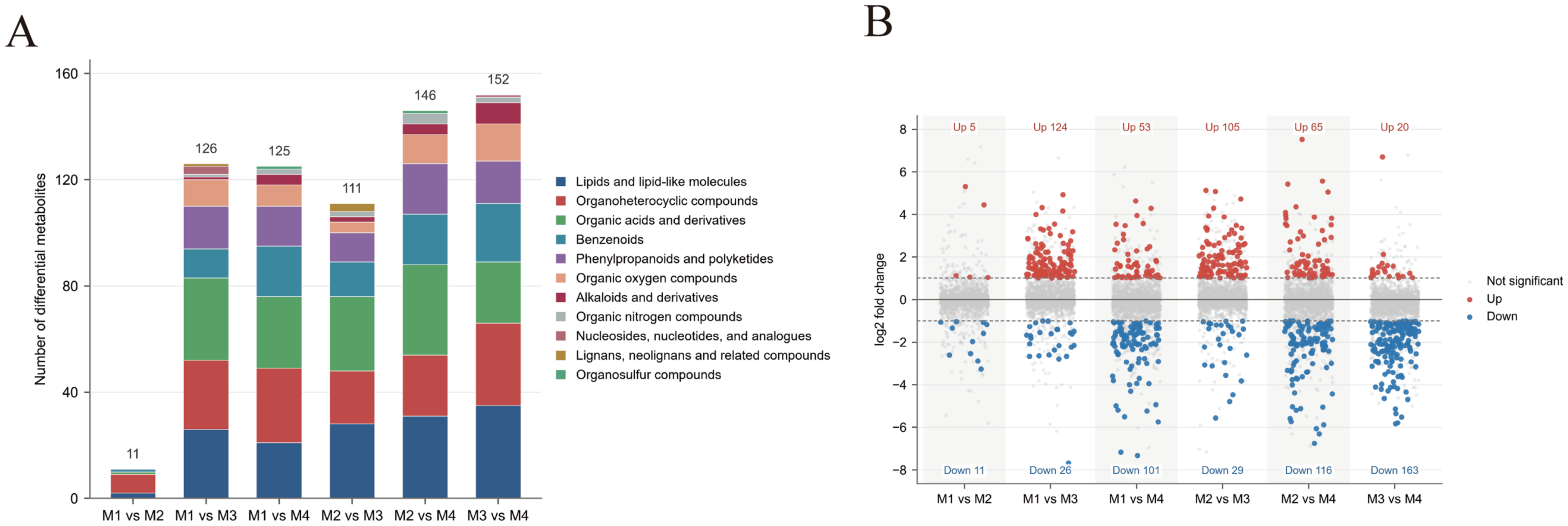
Differential non-volatile metabolites across six pairwise comparisons of fixation processing workflows. **(A)** Super_Class distribution of differential non-volatile metabolites with available Super_Class annotations. Unclassified metabolites were excluded from the class-distribution summary. **(B)** Volcano plots showing differential non-volatile metabolites screened using FDR < 0.05 and |log₂FC| > 1. M1, fully mechanical fixation; M2, mechanical–manual hybrid fixation; M3, manual–mechanical hybrid fixation; M4, fully manual fixation.

Differential non-volatile metabolites were screened using FDR < 0.05 and |log₂FC| > 1. The number of significantly differential non-volatile metabolites varied among the six pairwise comparisons: 16 in M1 vs. M2, 150 in M1 vs. M3, 154 in M1 vs. M4, 134 in M2 vs. M3, 181 in M2 vs. M4, and 183 in M3 vs. M4 (Figure 3B). The smallest difference was observed between M1 and M2, whereas comparisons involving M3 or M4 generally showed larger numbers of differential metabolites. This pattern was consistent with the global separation observed in the PCA plots.

The Super_Class distribution of differential non-volatile metabolites was further summarized using only metabolites with available Super_Class annotations. Under this classified-only framework, 11, 126, 125, 111, 146, and 152 classified differential metabolites were detected in M1 vs. M2, M1 vs. M3, M1 vs. M4, M2 vs. M3, M2 vs. M4, and M3 vs. M4, respectively (Figure 3A). The major contributing classes included lipids and lipid-like molecules, organoheterocyclic compounds, organic acids and derivatives, benzenoids, and phenylpropanoids and polyketides.

This class-level distribution provides chemical context for the non-volatile response to different fixation workflows. Lipids and lipid-like molecules may reflect changes in heat-sensitive membrane lipids and lipid-derived precursors, which can influence mouthfeel and provide substrates for downstream volatile formation. Phenylpropanoids and polyketides, benzenoids, and organic acids include many tea quality-related compounds, such as catechin derivatives, flavonoids, phenolic acids, and gallate-containing metabolites, which are closely associated with bitterness, astringency, and aftertaste. Organoheterocyclic compounds include alkaloid- and nitrogen-containing metabolites, some of which contribute to bitterness and thermal-reaction products. Therefore, the dominance of these classes indicates that the four workflows mainly affected metabolite pools linked to taste formation, phenolic transformation, lipid-derived reactions, and secondary metabolism, rather than producing random chemical variation.

**Figure 4:**
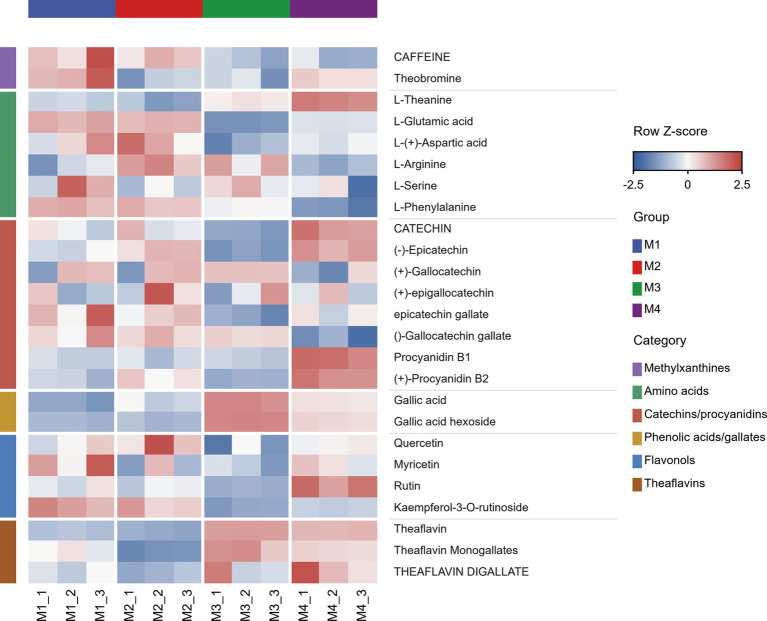
Abundance patterns of representative taste-associated non-volatile metabolites. Heatmap of 25 representative taste-associated metabolites selected based on putative annotation, literature-supported relevance to tea taste, acceptable QC performance, and coverage of major taste-related chemical groups. The color scale indicates scaled relative abundance across the four fixation processing workflows.

To further examine compounds with clearer taste relevance, 25 representative taste-associated non-volatile metabolites were selected from the QC-filtered LC-MS/MS dataset based on their putative annotations, literature-supported relevance to tea taste, acceptable QC performance, and coverage of major taste-related chemical groups, including catechins/procyanidins, amino acids, flavonols, theaflavins, methylxanthines, and phenolic acids/gallates (Figure 4). These metabolites showed workflow-dependent abundance patterns across M1–M4. Amino acids, catechin-related compounds, methylxanthines, phenolic acids, and theaflavin-related compounds contributed differently to the four chemical profiles. Several taste-associated metabolites, including L-phenylalanine, gallic acid hexoside, theaflavin, and theaflavin monogallates, also met the FDR-based differential screening criteria in at least one pairwise comparison. Because sensory evaluation was not performed, these compounds were interpreted as taste-associated chemical markers rather than direct evidence of perceived taste quality.

### 3.3 Differential volatile metabolites and candidate aroma-active compounds

**Figure 5:**
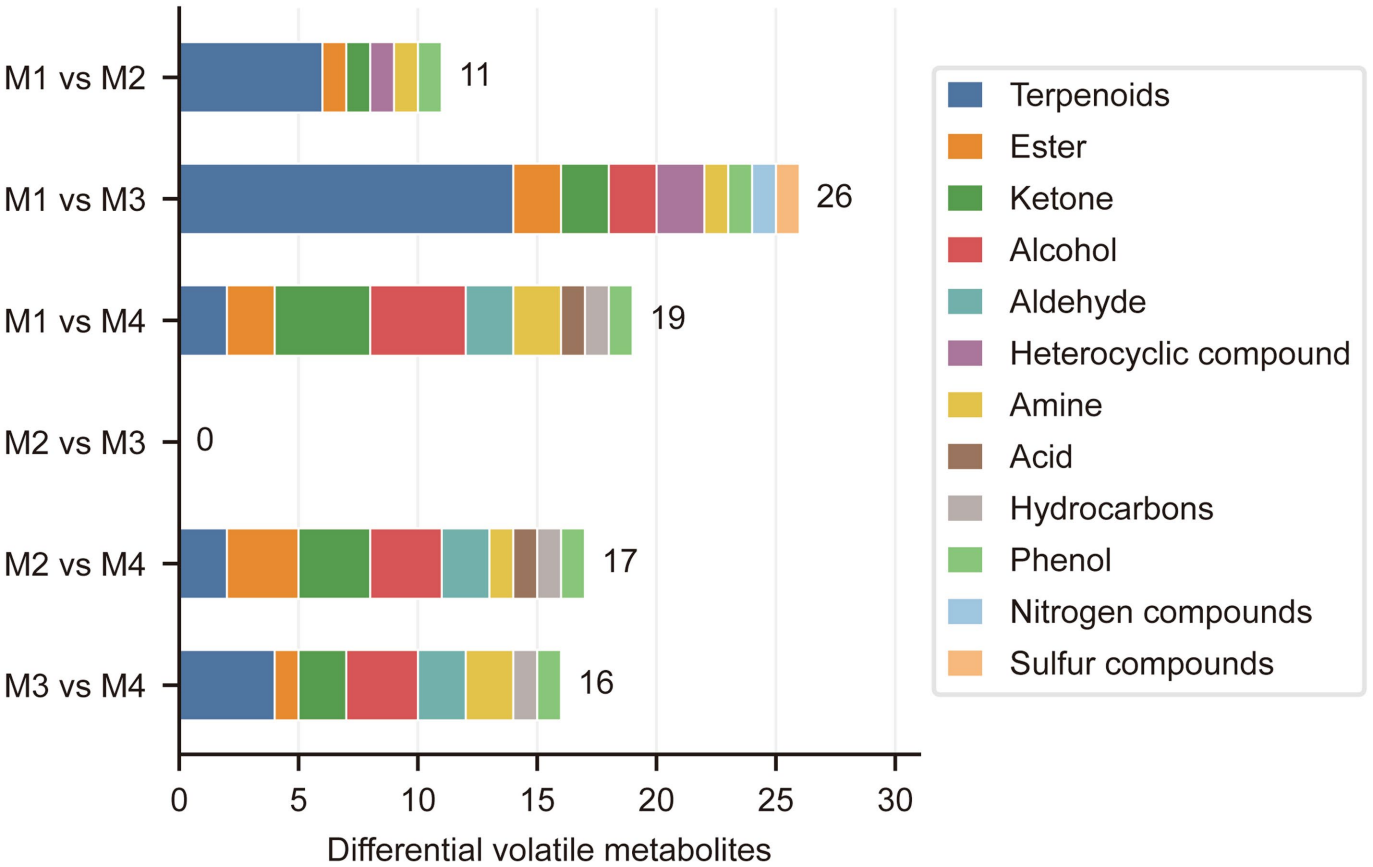
Differential volatile metabolites across six pairwise comparisons of fixation processing workflows. Differential volatile metabolites were screened using FDR < 0.05 and |log₂FC| > 1. The figure summarizes the number and chemical-class distribution of significantly differential volatile metabolites in each comparison. M1, fully mechanical fixation; M2, mechanical–manual hybrid fixation; M3, manual–mechanical hybrid fixation; M4, fully manual fixation.

Volatile metabolites were screened using FDR < 0.05 and |log₂FC| > 1. The numbers of significantly differential volatile metabolites were 11 in M1 vs. M2, 26 in M1 vs. M3, 19 in M1 vs. M4, 0 in M2 vs. M3, 17 in M2 vs. M4, and 16 in M3 vs. M4 (Figure 5). The absence of significant differential volatiles in M2 vs. M3 suggests that the two hybrid workflows produced relatively similar volatile profiles under the current statistical threshold.

The chemical-class distribution of differential volatiles showed that volatile changes were distributed across several aroma-related classes, including terpenoids, esters, ketones, alcohols, aldehydes, and heterocyclic compounds (Figure 5). Compared with the LC-MS dataset, the number of statistically differential volatile metabolites was smaller. This indicates that the volatile metabolome was either more conserved across workflows or more strongly constrained by the strict FDR threshold.

**Figure 6:**
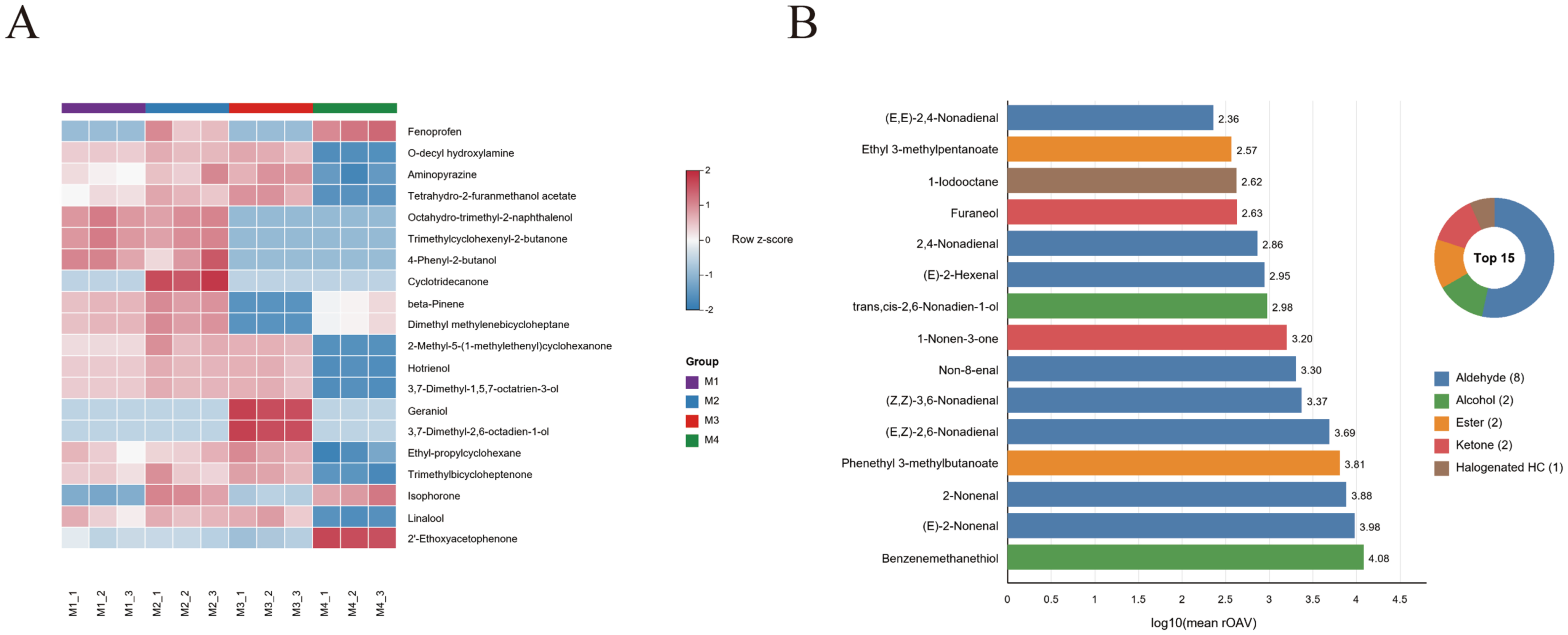
Differential volatile markers and candidate aroma-active compounds. **(A)** Heatmap of the top 20 differential volatile metabolites selected from 46 unique FDR-significant volatiles according to the maximum absolute log₂FC across pairwise comparisons. **(B)** Top 15 candidate aroma-active volatiles ranked by mean relative odor activity value (rOAV). Compound classes are indicated to show the chemical distribution of high-rOAV volatiles. Short labels are used for readability; full putative compound annotations are provided in Supplementary Table S2.

To identify volatile markers with stronger discriminatory patterns, the top 20 differential volatile metabolites were selected from 46 unique FDR-significant volatiles according to their maximum absolute log₂FC across pairwise comparisons (Figure 6A). These compounds separated the processing workflows into distinct abundance patterns and highlighted a subset of volatiles that contributed strongly to inter-group differences.

Candidate aroma-active volatiles were further prioritized using relative odor activity value (rOAV) ranking. The top 15 mean-rOAV compounds were dominated by aldehydes, together with selected alcohols, esters, ketones, and one halogenated hydrocarbon (Figure 6B). Highly ranked compounds included benzenemethanethiol, 2-nonenal isomers, 2,6-nonadienal isomers, non-8-enal, 1-nonen-3-one, trans,cis-2,6-nonadien-1-ol, (E)-2-hexenal, furaneol, and ester-type volatiles. These compounds are associated in published odor databases with descriptors such as green, fatty, cucumber-like, floral, fruity, sweet, and sulfur-like notes. However, because GC-O, aroma recombination, and sensory evaluation were not performed, these results should be interpreted as candidate aroma-related chemical evidence rather than confirmed sensory outcomes.

**Figure 7:**
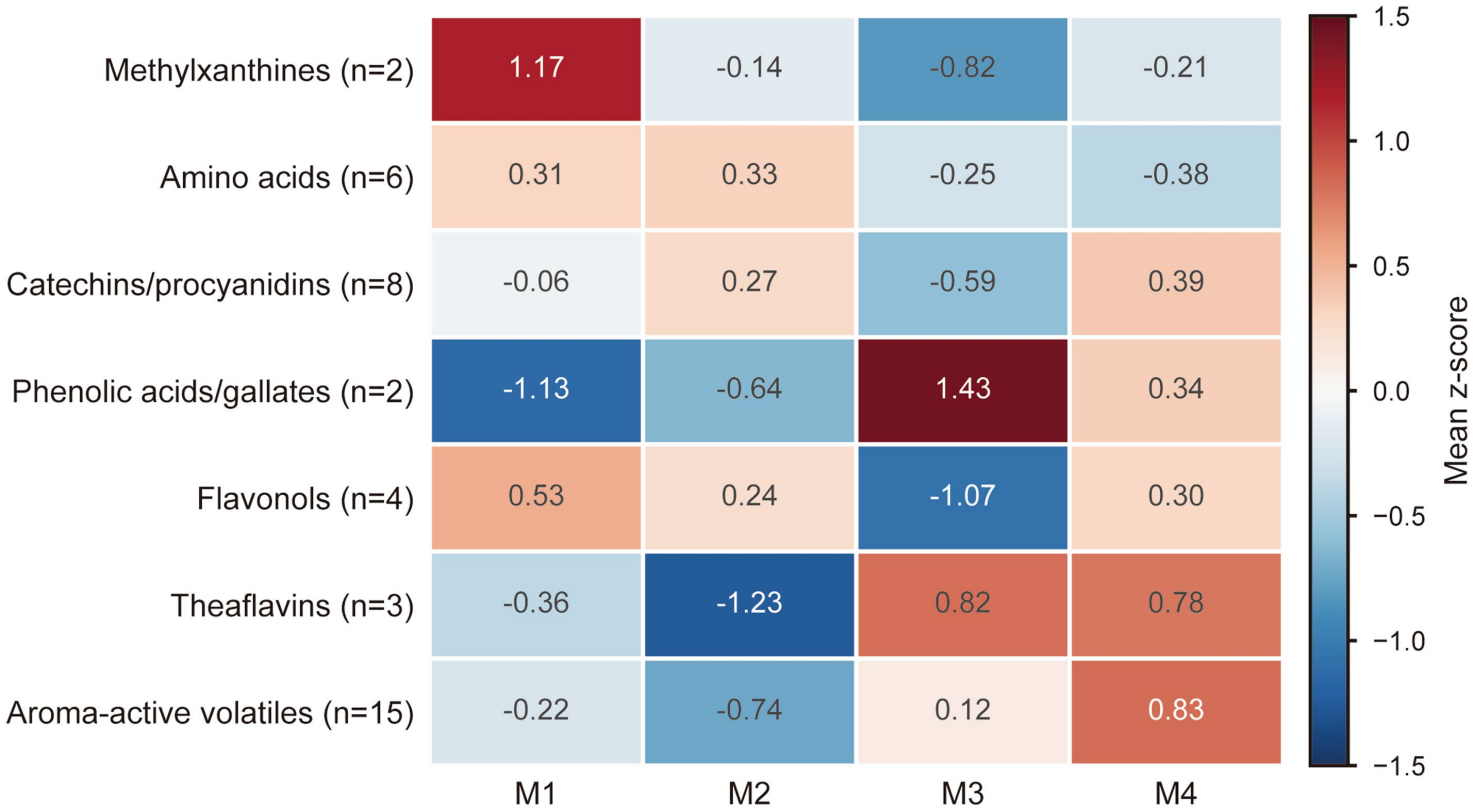
Integrated category-level patterns of taste-associated non-volatile metabolites and candidate aroma-active volatiles. Heatmap showing category-level abundance patterns of representative taste-associated non-volatile metabolite groups and rOAV-ranked volatile markers across the four fixation processing workflows. Values are shown as scaled relative abundance.

Finally, an integrated category-level heatmap was constructed to compare representative taste-associated non-volatile categories and aroma-active volatile markers across the four workflows (Figure 7). Methylxanthines showed relatively higher abundance in M1, phenolic acids/gallates were more prominent in M3, theaflavin-related compounds were higher in M3/M4, and aroma-active volatile markers showed the highest overall category-level signal in M4. Together, these results indicate that different fixation processing workflows produced distinct but partly overlapping chemical profiles, affecting both taste-associated non-volatile metabolites and candidate aroma-active volatiles.

## 4 Discussion

### 4.1 Integrated fixation histories redirect the metabolomic trajectory of Gougunao tea

The present study shows that Gougunao tea responds to fixation as an integrated thermal-mechanical history rather than as a single isolated processing step. The four protocols differed in fixation order, heating intensity, exposure time, and rolling duration. These variables jointly determine enzyme inactivation, tissue disruption, dehydration, and the duration of heat-driven metabolite transformation. Therefore, the chemical separation observed among M1–M4 should be interpreted as the outcome of different fixation–rolling trajectories rather than the effect of fixation sequence alone (4, 8–10, 14).

The first fixation step appeared to exert a strong organizing effect on the final non-volatile profile. M1 and M2 both began with high-temperature short-time mechanical fixation and showed the smallest number of differential non-volatile metabolites. This pattern suggests that rapid early heating may quickly restrict residual enzymatic oxidation and hydrolysis, thereby setting a relatively stable initial chemical state. In contrast, M3 and M4 began with lower-temperature but longer-duration manual fixation. This slower thermal input may extend the transitional period in which residual enzymatic activity, tissue disruption, dehydration, and thermal conversion occur together, providing a plausible basis for the larger metabolomic shifts observed in comparisons involving M3 and M4 (2, 5, 7).

The supervised models further supported chemical discrimination among the four protocols. However, because each treatment contained three biological replicates, OPLS-DA was used as evidence of group separation rather than as independent proof of mechanism. The main biological interpretation was therefore based on FDR-controlled differential metabolites, with the multivariate models serving to summarize global chemical structure (15, 16). This distinction is important because metabolomic separation indicates reproducible chemical patterning within the experiment, whereas mechanistic inference requires interpretation through known tea-processing chemistry.

### 4.2 Non-volatile changes reflect competing preservation and transformation of taste-related pools

The LC-MS results indicate that fixation mainly reshaped metabolite pools associated with tea taste and secondary metabolism. Phenylpropanoids and polyketides, benzenoids, organic acids, gallate-containing compounds, amino acids, methylxanthines, and lipid-like molecules accounted for much of the differential signal. These classes are chemically connected through oxidation, hydrolysis, conjugation, thermal degradation, and precursor-product relationships. Their coordinated changes suggest that fixation altered the balance among bitterness, astringency, umami, aftertaste, and mouthfeel-related components rather than simply changing total metabolite abundance (17–19).

Phenolic and gallate-related metabolites were especially informative for interpreting manual-involved protocols. Catechins, flavonoids, phenolic acids, and theaflavin-related compounds are sensitive to both enzymatic and thermal transformation during tea processing. The stronger phenolic acid/gallate signal observed in M3 may reflect partial hydrolysis or conversion of gallated catechins during the longer early manual fixation and rolling stage. In this setting, the subsequent mechanical fixation may terminate reactions after a longer transformation window has already occurred. By contrast, immediate mechanical fixation in M1 and M2 may restrict these conversions earlier (2, 7, 12).

Amino acids and methylxanthines provide a second layer of taste relevance. Free amino acids, including theanine, glutamine, phenylalanine, and related compounds, contribute to umami, sweetness, and taste balance. Caffeine and related methylxanthines contribute bitterness. Their distinct abundance patterns indicate that the four protocols shifted the ratio among taste-associated chemical groups. This is important because green tea taste is usually determined by the balance among compound classes rather than by one dominant metabolite (17, 18).

Lipids and lipid-like molecules also contributed substantially to the LC-MS differential profiles. Although these compounds are not usually discussed as direct taste compounds in tea infusion, they are sensitive to heat, dehydration, and tissue disruption. Lipid degradation can influence mouthfeel and provide precursors for aldehydes, ketones, alcohols, and esters. Thus, lipid-related LC-MS changes may represent an upstream chemical connection between non-volatile metabolite remodeling and volatile aroma formation (20, 21).

### 4.3 Volatile differences point to distinct routes of aroma precursor conversion

The GC-MS results showed fewer FDR-significant differences than the LC-MS dataset, indicating that the volatile fraction was more selective in its response to processing. This does not mean that aroma chemistry was unaffected. Volatile formation depends on multiple coupled processes, including precursor availability, release from bound forms, lipid oxidation, amino acid degradation, and thermal reactions. These pathways can converge across manufacturing routes even when the broader non-volatile metabolome remains distinct (21–23).

The absence of significant volatile differences between M2 and M3 is therefore chemically informative. Both protocols combined mechanical and manual fixation, but in opposite orders. Their similar volatile profiles suggest that hybrid thermal-mechanical histories may converge toward a comparable aroma precursor balance, even when their non-volatile matrices remain more strongly separated. In contrast, the stronger volatile signals in comparisons involving M4 suggest that prolonged manual fixation and longer rolling may favor the accumulation or release of aroma-active compounds.

Aldehydes, esters, alcohols, ketones, terpenoids, and heterocyclic compounds dominated the differential volatile profiles, whereas the rOAV-ranked compounds were mainly aldehydes, with additional alcohols, esters, ketones, and one halogenated hydrocarbon. C6 and C9 aldehydes are commonly linked to lipid oxidation and green or fatty notes, whereas terpenoids and esters are often associated with floral and fruity attributes. Although sulfur-containing compounds were not treated as a separate chemical class in the figure, benzenemethanethiol ranked highest by mean rOAV and is associated with sulfur-like, garlic-like, onion-like, and coffee-like descriptors. These high-rOAV compounds can strongly influence aroma even at low abundance (21–23). The higher category-level aroma-active signal in M4 is therefore consistent with a manufacturing route that combines longer thermal exposure, stronger manual handling, and extended opportunity for precursor conversion.

These results do not necessarily imply that one protocol produced superior sensory quality. A more defensible interpretation is that each thermal-mechanical history directed Gougunao tea toward a different chemical style. Mechanical-dominant processing appeared to preserve a more rapidly fixed profile, whereas manual-dominant processing promoted broader transformation of phenolic, lipid-derived, and aroma-related metabolites. Taken together, the volatile data suggest that while the non-volatile matrix is strongly influenced by the first fixation step, the final aroma profile is more tightly constrained by cumulative thermal input across the entire workflow (21–25).

### 4.4 Implications for processing control and chemical quality design

These findings position fixation as a chemical control point for steering Gougunao tea quality. By selecting mechanical, manual, or hybrid protocols, producers may differentially modulate chemical drivers of phenolic astringency, amino acid-associated umami, methylxanthine-related bitterness, and lipid-derived aroma precursors. Thus, fixation design does not merely determine enzyme inactivation efficiency. It also reconfigures the chemical balance among taste-associated non-volatile metabolites and aroma-associated volatile markers (6, 13, 17–23).

This metabolomic framework is useful for process evaluation because it extends beyond single-compound comparisons of finished teas. The integrated LC-MS/MS and GC-MS strategy identified which chemical groups were most responsive to thermal-mechanical variation and how different manufacturing routes produced partly overlapping but distinguishable chemical fingerprints. Such information can guide targeted optimization of Gougunao tea processing, especially when producers aim to balance phenolic transformation, bitterness/astringency, umami-related compounds, and aroma precursor conversion.

Several boundaries should be considered. Most LC-MS/MS annotations were MSI Level 2 putatively annotated features, and the GC-MS results were based on SIM-supported relative quantification. In addition, no sensory evaluation, GC-O analysis, or aroma recombination experiment was performed. Therefore, the chemical patterns reported here should be interpreted as workflow-associated metabolomic evidence rather than direct sensory quality ranking. The experiment also used three biological replicates from one homogeneous fresh-leaf batch, so these findings represent a controlled-batch chemical map rather than a universal ranking of all Gougunao tea products (15, 16, 26, 27). Future work should test whether the same trajectories are reproduced across seasons, gardens, and production scales, and should combine targeted quantification with sensory and aroma-validation experiments.

Overall, the results reveal that Gougunao tea fixation reshapes quality-related chemistry through coordinated changes in non-volatile taste-associated metabolites and volatile aroma-associated markers. The distinction among mechanical, manual, and hybrid protocols provides a chemical basis for understanding how thermal-mechanical processing choices shape the final metabolomic profile of Gougunao green tea.

## 5 Conclusion

This study used integrated LC-MS/MS and GC-MS metabolomics to characterize how mechanical, manual, and hybrid fixation protocols shape the chemical profile of Gougunao green tea. The four thermal-mechanical processing histories produced distinguishable non-volatile and volatile metabolomic patterns, indicating that fixation design affects tea chemistry beyond simple enzyme inactivation.

The LC-MS/MS results showed that fixation mainly reshaped taste- and secondary-metabolism-related pools, including phenolic compounds, gallate-related metabolites, amino acids, methylxanthines, organic acids, and lipid-like molecules. These changes suggest that different fixation protocols altered the chemical balance associated with bitterness, astringency, umami, aftertaste, mouthfeel, and volatile precursor formation. The GC-MS results further showed that volatile differences were concentrated in aroma-relevant compounds, including aldehydes, esters, alcohols, ketones, terpenoids, heterocyclic compounds, and sulfur-containing volatiles. Together, these results reveal coordinated changes between non-volatile taste-associated metabolites and volatile aroma-associated markers.

Overall, fixation can be regarded as a key chemical control point in Gougunao tea manufacturing. Mechanical-dominant, manual-dominant, and hybrid protocols did not simply produce stronger or weaker versions of the same chemical profile, but directed the tea toward different metabolomic states. This study provides a chemical basis for optimizing Gougunao tea processing through targeted control of thermal-mechanical history. Future validation across multiple batches, seasons, and production scales, combined with targeted quantification and sensory evaluation, will help translate these metabolomic patterns into practical quality-control strategies.

## Data Availability

The raw LC-MS/MS and GC-MS datasets generated during this study have been deposited in the MetaboLights repository under the accession number MTBLS15065 and are publicly available at https://www.ebi.ac.uk/metabolights/MTBLS15065. All processed metabolomics datasets, including metabolite identification and quantification tables, are provided as Supplementary Tables S1 and S2 to support the findings of this study. Please contact the corresponding author at jgsdxzwzy@hotmail.com for any additional data access requests.

## Supplementary material

SUPPLEMENTARY FIGURE S1 Pairwise OPLS-DA score plots based on the LC-MS dataset. The six pairwise OPLS-DA score plots show supervised discrimination among the four fixation processing workflows based on the LC-MS non-volatile metabolomic dataset. M1, fully mechanical fixation; M2, mechanical–manual hybrid fixation; M3, manual–mechanical hybrid fixation; M4, fully manual fixation.

SUPPLEMENTARY FIGURE S2 Permutation tests of the LC-MS OPLS-DA models. Permutation tests were used to assess potential overfitting of the six pairwise LC-MS OPLS-DA models. The original Q² values were compared with the permuted Q² distributions generated from 200 permutation iterations.

SUPPLEMENTARY FIGURE S3 Pairwise OPLS-DA score plots based on the GC-MS dataset. The six pairwise OPLS-DA score plots show supervised discrimination among the four fixation processing workflows based on the GC-MS volatile metabolomic dataset. M1, fully mechanical fixation; M2, mechanical–manual hybrid fixation; M3, manual–mechanical hybrid fixation; M4, fully manual fixation.

SUPPLEMENTARY FIGURE S4 Permutation tests of the GC-MS OPLS-DA models. Permutation tests were used to assess potential overfitting of the six pairwise GC-MS OPLS-DA models. The original Q² values were compared with the permuted Q² distributions generated from 200 permutation iterations.

SUPPLEMENTARY TABLE S1 LC-MS non-volatile dataset. Sheet1 contains the curated and annotated LC-MS non-volatile metabolite table used for downstream analysis in this study, including metabolite IDs, retention time, m/z, annotation information, chemical classification, annotation level, peak areas across samples and QC samples, and QC RSD values. POS and NEG contain the full positive- and negative-ion feature tables before final curation, including complete peak-area information and QC metrics.

SUPPLEMENTARY TABLE S2 GC-MS volatile dataset. Sheet1 contains the curated, analysis-ready GC-MS volatile peak-area table used in this study. This table was derived from Sheet2 after data cleaning, with nondetected peaks imputed as one-fifth of the row-wise minimum positive value and with the annotation field standardized where applicable. Sheet2 contains the original GC-MS peak-area matrix exported from data processing, in which nondetected peaks were retained as missing values.
